# Home Monitoring System for Comprehensive Geriatric Assessment in Patient's Dwelling: System Design and UX Evaluation

**DOI:** 10.3389/fdgth.2021.659940

**Published:** 2021-05-07

**Authors:** Elena Villalba-Mora, Xavier Ferre, Rodrigo Pérez-Rodríguez, Cristian Moral, Myriam Valdés-Aragonés, Alberto Sánchez-Sánchez, Leocadio Rodríguez-Mañas

**Affiliations:** ^1^Center for Biomedical Technology, Universidad Politécnica de Madrid, Madrid, Spain; ^2^Biomedical Research Networking Center in Bioengineering Biomaterials and Nanomedicine (CIBER-BBN), Madrid, Spain; ^3^Biomedical Research Foundation, Getafe University Hospital, Getafe, Madrid, Spain; ^4^Getafe University Hospital, Getafe, Madrid, Spain

**Keywords:** older population, home monitoring, frailty, user experience, usability, acceptability, active aging

## Abstract

Population aging threatens the sustainability of welfare systems since it is not accompanied by an extended healthy and independent period in the last years of life. The Comprehensive Geriatric Assessment (CGA) has been shown to be efficient in maintaining the healthy period at the end of the life. Frailty monitoring is typically carried out for an average period of 6 months in clinical settings, while more regular monitoring could prevent the transition to disability. We present the design process of a system for frailty home monitoring based on an adapted CGA and the rationale behind its User eXperience (UX) design. The resulting home monitoring system consists of two devices based on ultrasound sensors, a weight scale, and a mobile application for managing the devices, administering CGA-related questionnaires, and providing alerts. Older users may encounter barriers in their usage of technology. For this reason, usability and acceptability are critical for health monitoring systems addressed to geriatric patients. In the design of our system, we have followed a user-centered process, involving geriatricians and older frail patients by means of co-creation methods. In the iterative process of design and usability testing, we have identified the most effective way of conducting the home-based CGA, not just by replicating the dialogue between the physician and the patient, but by adapting the design to the possibilities and limitations of mobile health for this segment of users. The usability evaluation, carried out with 14 older adults, has proved the feasibility of users older than 70 effectively using our monitoring system, additionally showing an intention over 80% for using the system. It has also provided some insights and recommendations for the design of mobile health systems for older users.

## Introduction

Aging populations in developed countries threaten the sustainability of welfare systems. The population segment aged 65 or older is growing both in absolute terms and as percentage of the total (1). However, the increase in longevity is not accompanied by an extended healthy and independent period in the last years of life, which entails negative and burdensome implications for societies in the coming years (2). Many older adults suffer from multiple chronic diseases and conditions (3). In addition, organ malfunction and the lack of physiological reserves may lead to the onset of frailty (4). Frailty is a stage preceding disability in which the intrinsic capacity of patients declines, increasing their vulnerability to stressors and the risk of sudden catastrophic deterioration in health and function (5).

Geriatric medicine promotes active aging, fostering a proactive and predictive care approach to the management of older adults (6). One of their pillars is the maintenance of patients' functionality and independence through the prediction and prevention of adverse events and the resulting impairing or disabling consequences (7). The Comprehensive Geriatric Assessment (CGA) proposes a multidimensional evaluation of patients, including co-morbidities, cognitive, mental and functional status, social situation, polypharmacy and nutrition (8). CGA has the potential to reduce the slope of the aging trajectory, preventing the onset of frailty, and enabling not only a longer, but also a better life for older persons (9).

The implementation of a CGA depends upon several specialists from different care levels. Additionally, completing the CGA requires time and personnel resources, which in some cases are not readily available for assessing the whole population at risk of frailty (10). Alternative CGA models have been proposed to overcome this barrier, which include an abbreviated version of CGA (11); or the incorporation of Information and Communication Technologies (ICT) to integrate all professionals and care levels involved in the assessment, and to streamline data collection and patient management (12). Indeed, ICT does not only allow remote gathering of relevant information, and the generation and integration of knowledge, it may also empower patients to actively participate in the management of their own health and transform the traditional relationship between patients and professionals (13). Furthermore, it lightens the workload on professionals, enhances the ubiquity of care, and provides all specialists involved with decision-supporting information. As a result, the World Health Organization has recently stated that “there is a pressing need to develop comprehensive community-based approaches and to introduce interventions to prevent declining capacity and provide support to informal caregivers” (14).

Mobile health (mHealth) supports the provision of healthcare services ubiquitously (15). According to Searcy et al. the adoption of this technology by the older population is still low in comparison to other groups, which compromises the applicability and generalization of mHealth-supported CGA strategies (16). The causes of this phenomenon range from physical barriers to patients' thoughts and attitudes toward technology (17). Physical impairments associated with aging, including vision, hearing, and proprioceptive decline, affect the UX of usage of ICT systems. But these obstacles do not preclude older users from using ICT, if they have enough motivation and the system is designed taking into account the characteristics of this kind of user. A user-centered, collaborative, and interdisciplinary approach may enhance feasibility, acceptability, and usability of mHealth solutions for older adults (18).

**Figure 1:**
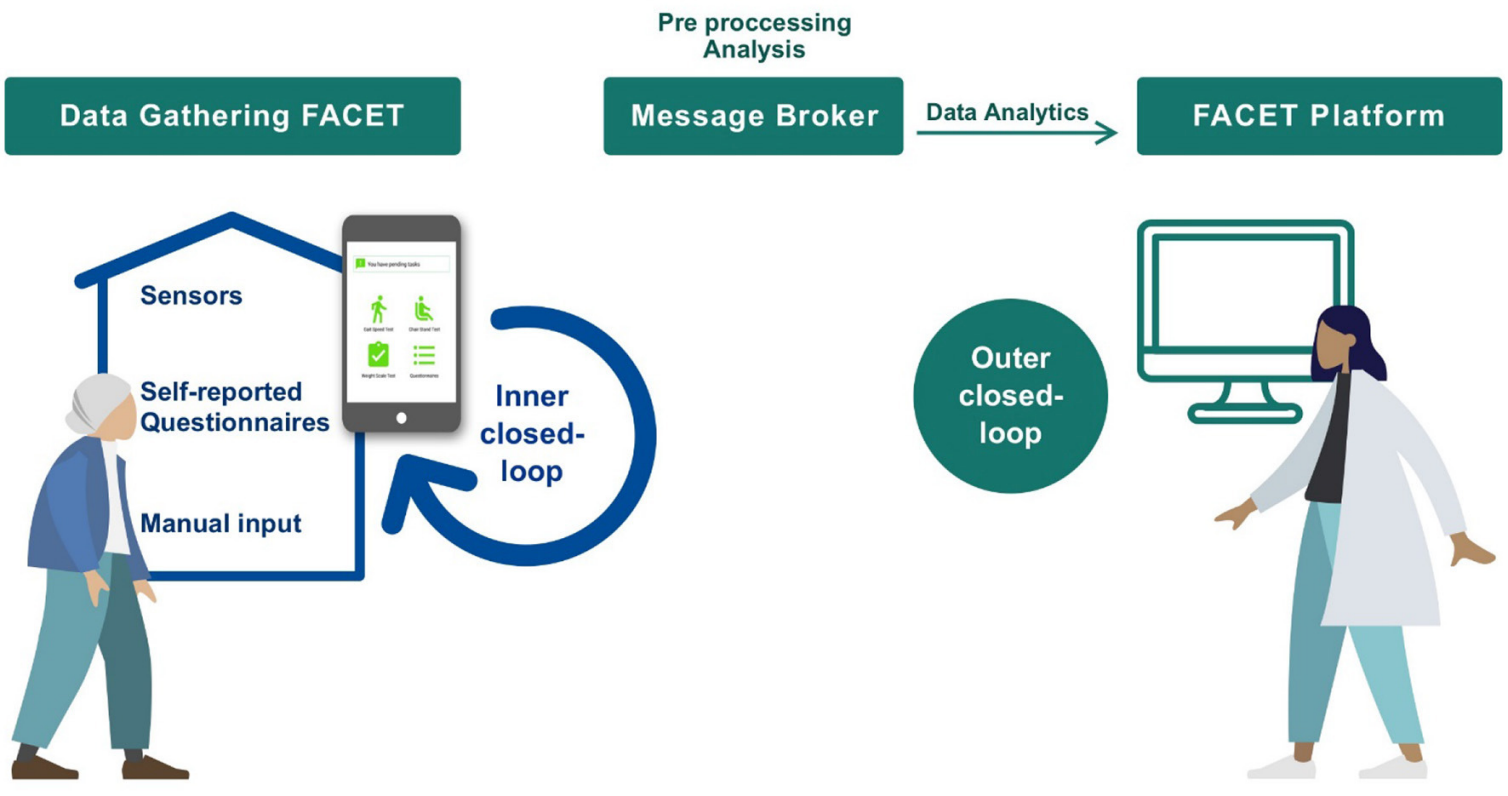
Description of the FACET system that incorporates our HMS.

This paper presents the design and UX evaluation of a Home Monitoring System (HMS) that supports the CGA in a patient's dwelling. Our HMS is designed to be linked to the Integrated Care Programme for older adults, which has been implemented at the University Hospital of Getafe for more than 25 years (19). In this manner, the European Project FACET (Integrated supportive services/products to promote FrAilty Care and wEll funcTion) brings care to the home to prevent, detect, monitor, and ultimately better manage frailty. The resulting system includes the HMS presented in this paper, that is, connected to a service platform which stores the gathered data and raises alarms for the clinical professional. Alarms are generated when there is a deterioration in the patient's frailty status according to the clinical guidelines. An outline of the overall FACET scheme is shown in Figure 1.

The HMS consists of two sensor-based devices designed by the research team from Universidad Politécnica de Madrid, a commercial smart weight scale, and a mobile application. Thanks to this data gathering kit, health care professionals can collect relevant information from patients by monitoring their performance in activities that are relevant to CGA. In our solution, usability and acceptability of the proposed technology are key elements to ensure later adoption. To reach this goal, we have followed a user-centered approach where participatory design and UX evaluation results have guided the three design iterations carried out as proposed by Merkel and Kucharski (20).

The following section presents the methods followed. Section Design of the Home Monitoring System presents the design of the HMS. Later, section Results presents the results of the usability tests and the resulting improvements. Section Discussion discusses the results. Finally, section Conclusions and future work summarizes the conclusions reached and the future research.

## Methods

The design of any mHealth system to be used outside a clinical setting is a challenge, as it requires good UX to ensure patients' acceptability and adherence. When the target users of the system are older persons, the challenge is even greater due to the specific limitations in their interactions with computing systems. We adopted a User-Centered Design (UCD) approach (21) to ensure that the usability and acceptability of the solution would provide an appropriate UX. UCD is an iterative process which includes the following activities: (a) analysis and specification of the context of use, (b) user requirements, (c) development of solutions and (d) usability testing. To reach this goal, we carried out three iterations until the results of the usability testing were acceptable for use by our older patients at home:

1. In the first iteration, the main objective was to develop a deep understanding of the characteristics of the users and its actual context of use. We adopted a participatory design, which is the part of the co-creation process that focuses on design activities (22) and in which users are actively involved in the design. The healthcare professionals of the Geriatrics Unit at University Hospital of Getafe (HUG) in Madrid participated, contributing to the analysis and specification of the context of use, and to the first design of the HMS. First, we conducted a contextual inquiry observation at the hospital, and we interviewed the professionals to characterize how CGA is carried out with patients in the clinical practice. Second, the results were taken to a focus group session using scenarios and personas to facilitate communication among all participants. Third, we then carried out a brainstorming co-design session to create the initial wireframes of the system. Fourth, a low-fidelity prototype was created based on these wireframes and was refined together with the healthcare professionals using a cognitive walkthrough (23).
2. In the second iteration, we developed and tested a high-fidelity prototype to run on a tablet connected to monitoring devices. The prototype included all the interactions and visual graphics required to resemble a finalized system. We then assessed the UX.
3. Finally, in the third iteration, we improved the prototype by resolving the issues detected previously. Then, we performed a second evaluation, again assessing UX to compare the results obtained in both tests.

During the second and third iterations, we assessed the UX of the HMS by evaluating the usability and acceptability of the mobile app and the devices with older adults in their homes.

To analyze the usability, we measured three attributes as specified in the ISO 9241-210:2019 (21): effectiveness, efficiency, and satisfaction. Participants were asked to perform four predefined tasks, as described in section Results. Effectiveness was assessed via the rate of tasks completed by the participants and the average rate of errors. Participants were asked to complete the tasks without asking for help, except if they were stuck and could not finish the task on their own. In this case, the user was provided with minimal guidance to progress, and the task was registered as “finished with help.” If the user could not finish the task even with this minimal guidance, it was registered as “unable to finish the task.”

Efficiency was measured via the difference between the average number of taps made by participants to complete a task and the optimal number made by the designers. For satisfaction, we used the System Usability Scale (SUS) questionnaire (24), that provides satisfaction perceived by the user after interacting with the system in terms of willingness to use the system daily, ease of use, learnability, and internal consistency. In addition, participants were asked to use the Think Aloud protocol (25) to collect all their comments and impressions while using the system, and observers registered any relevant observations or user remarks.

In addition to usability, acceptability was measured using the questionnaire proposed by Villalba et al. (26), that consists of three open questions assessing the acceptability of the HMS in general, and a set of 4 Likert-based questions individually assessing each of the components of the system—in our case, the sensor-based devices. Based on the answers provided to the Likert-based questions, it is also possible to calculate a score reflecting the level of acceptability. These questionnaires are detailed in Appendix A.

Ethical approval was requested and obtained from the Clinical Research Ethics committee of the Getafe University Hospital on October 29, 2017, with number 17/72. Participants were recruited by the geriatrician participating in the study, according to the following eligibility criteria:

- Inclusion criteria: (1) age ≥ 70 years old; (2) participants must live in their own homes, not in a nursing homeor residence; (3) with family support; and (4) ability to walk with or without technical assistance.
- Exclusion criteria: (1) inadequate home infrastructure for the installation of the sensor-based devices; (2) participant's inability to understand and use the HMS; (3) any medical illness that makes it impossible to perform physical exercises and the scheduled tasks (Acute myocardial infarction in the last 3 months, Unstable cardiovascular disease, Terminal illness, Other pathologies involving clinical instability, at the discretion of the clinician); (3) previous functional impairment resulting in dependency (Barthel Index <40); (4) history of alcohol and/or drug abuse; and (5) psychiatric disorders (schizophrenia, psychotic disorders).

## Design of the Home Monitoring System

This section describes the design of the HMS which is part of the overall FACET system (Figure 1). In FACET, the proposed care model incorporates patients from the geriatric services at the hospital as an entry point into the overall system. When a patient is at risk of frailty, he can be granted a program that includes the remote CGA assessment. The clinical team explains to the patient how the system works. Later, the technical team in charge of technical assistance, installs the HMS at home, calibrates the devices and trains the patient to use the different parts of the HMS. The patient's progress is followed by geriatricians through a web application.

During the first iteration of the UCD, we analyzed which parts of the CGA can be completed at home, based on clinical relevance, required periodicity, and the technical feasibility of unsupervised performance. Regarding the functional tests, we decided to omit the grip strength measurement, due to the lack of appropriate hardware for digital measurement at a reasonable cost, and the equilibrium tests, since there is a risk of falling for the patient when performing these tests unsupervised.

As a result, the tests selected for the remote assessment were: (1) the gait speed (27), (2) the 30-s sit-to-stand-test (also known as chair stand test) (28), (3) non-voluntary weight loss (29), (4) the Linda Fried's Frailty Criteria (5), (5) the Mini Nutritional Assessment (30), (6) the Barthel Index (31), (7) the Functional Activities Questionnaire (32), and (8) the FRAIL Scale (33). Thus, the HMS must include external devices to self-measure gait speed, the chair stand test, and weight; as well as an interaction device to assist the patient while self-measuring, and to answer to questionnaires.

**Figure 2:**
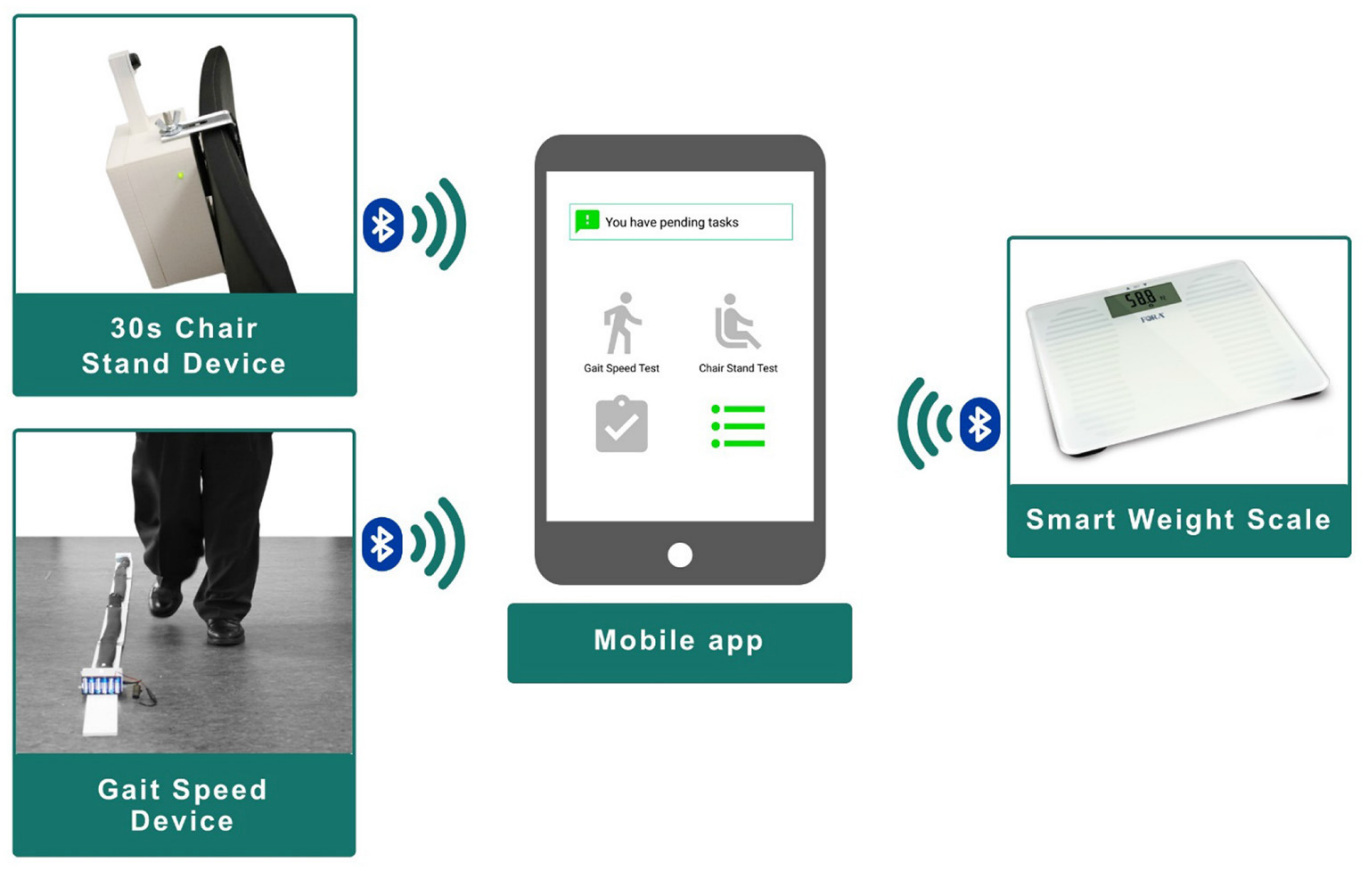
Home monitoring system.

For the chair stand test, we developed a lightweight and low-cost ultrasonic sensor-based system, which measures how many times the patient stands up from a chair in 30 s (34). The device must be fixed on top of a chair, comprising an ultrasound sensor, an Arduino board and a Bluetooth transmitter enclosed in a 3D-printed box. The device was designed and constructed at the Aging Lab at Center for Biomedical Technology at Universidad Politécnica de Madrid. The design and the results of the feasibility and effectiveness (assessed first, at the laboratory and later, at the University Hospital of Getafe with geriatric patients) are presented in Cobo et al. (35). The resulting device is shown in Figure 2 (top left picture).

To measure the gait speed, we also built the device upon a lightweight and low-cost ultrasonic sensor-based system. It was also designed and constructed at the Aging Lab. The device consists of two ultrasound sensors located in a straight line at 2.4 m from each other and connected to an Arduino board. The system integrates a Bluetooth transmitter to send the collected data wirelessly. The feasibility and effectiveness of the device was assessed first, at the laboratory and later, at the University Hospital of Getafe with geriatric patients. Results are presented in Ferré et al. (36). The resulting device is included in Figure 2 (bottom left picture).

Finally, the HMS also includes a commercial smart weight scale that sends the weight measures via Bluetooth (Figure 2, right).

During the observations, we witnessed that geriatricians adapted the words and language of the questionnaires for the older people. Therefore, we asked the geriatricians to adapt the questionnaires to be self-administered through our mobile app to make them more readable and understandable, ensuring that patients can independently answer them without needing external help. This involved simplifying the terminology, changing the subject of the questions to directly address the patients, dividing complex questions into simpler and shorter ones, and/or converting questions with several possible answers (e.g., Likert) into dichotomous “Yes/No” questions.

The adaptation of the questionnaire of the Linda Fried's Frailty Criteria, which was used in the evaluation, is presented in Appendix B.

We then created a first low-fidelity prototype for the interaction device which was tested in a cognitive walkthrough at the hospital. The main decisions that emerged from that session were:

- Regarding the preferred interaction device, even though the mobile app could be used on a smartphone, a tablet would be better for older adults due to the bigger screen size.
- Using both vertical and horizontal orientations was discarded, selecting only a vertical mode to avoid confusing the users not familiarized with rotating a mobile device.
- The user must be able to navigate among the screens, for example, when choosing which task to perform. This necessitates having “Back” and “Next” buttons on all the process screens.
- To avoid rejection and frustration, the process must be a guided experience, providing all the explanations in textual form, so that the users can access all the information and know what to do.
- To strengthen their confidence, users should be asked to confirm their actions.
- For completing the questionnaires, the form layout cannot be used as it is too confusing and complex for older users. Instead, questionnaire screens should consist of individual questions and short closed answers.

With those decisions, we programed a first high-fidelity prototype in Android. The mobile app acts as an interaction device, guiding the user in performing the different tasks and as a concentrator for the data coming from the monitoring devices via Bluetooth connection. For instance, when the patient must monitor his gait speed, the app will ask the user to first place correctly the device, later to switch it on, and when the connection is working, the app asks the user to walk between the ultrasound sensors. If the measurement is correctly received, the app shows it to the patient and then the user switches it off and gathers it up. If there is an error, the app will handle it and if necessary, it will raise an alert to the technical assistance team.

Section Results describes the second and third iteration, presenting usability testing and the implications for the final design of the mobile application which is illustrated in Figure 2 (center).

## Results

This section presents the results of the two tests we performed to assess the UX of the mobile application and the three external devices. First, we present the test procedure and the participants descriptive data. Later we present the results in terms of effectiveness, efficiency, satisfaction, and acceptability. To finish this section, we present the usability problems that we detected and how we improved them until participants were able to use adequately the HMS.

### Test Procedure and Participant Demographics

During the tests, we conducted the following process:

1. Upon arrival, the purpose of the study and the tasks to be accomplished were explained to the participants. Patients read and signed an informed consent containing this information, together with the ethical aspects of the study. Patients were encouraged to ask for any clarification, if needed, before signing. There were no foreseen risks in this study.
2. Participants were asked to fill out a demographic and clinical questionnaire. The answers were used only to ensure *in situ* that the eligibility criteria were met.
3. An initial training was provided to the participants to explain how the HMS works and what they were expected to do. In addition, they were handed a hardcopy with the instructions for each task they would be asked to carry out.
4. The participants were asked to perform four tasks: (1) Chair Stand test; (2) Adapted Linda Fried's questionnaire; (3) Gait Speed test; and (4) Weighing oneself. In the first iteration, each task started with a preconfigured reminder in the mobile application that prompted the user to perform a task.
5. During the performance of each task, the members of the research team acted as observers, registering user errors, task completion, number of taps, user remarks and observations of interest.
6. At the end of each task, participants were asked to answer the specific acceptability questionnaire about the device they had just operated.

It is worth mentioning that in tasks (1), (3), and (4), we were not only analyzing the app in the tablet, but also the operation of the devices connected to it, since the participants were asked to follow the instructions given by the app, including operating with the devices, placing them, switching them on, performing the measure, visualizing the results in the app, and, finally, switching them off.

Once all tasks were completed, participants were asked to fill in the SUS questionnaire, and the overall system acceptability questionnaire.

In total, 14 users participated: eight older users assessed the first high-fidelity prototype, whereas six participants tested the second one. Table 1 summarizes the demographic data of the participants in both usability tests. Females outnumber males, which reflects gender distribution in this age range. We can see a distribution of education levels among subjects, and all of them except one live with a same-age relative. All subjects but one have limited technology experience.

**Table 1:** Summary of the demographic data in the two rounds of UX evaluation.

	**First usability test**	**Second usability test**
**Age (mean)**	80	77.17
**Age (standard deviation)**	4.63	3.78
**Gender**		
Male	3 (38%)	2 (33%)
Female	5 (62%)	4 (67%)
**Education**		
Without regulated education	3 (37.5%)	2 (33.33%)
Primary	2 (25%)	1 (16.67%)
Secondary	3 (37.5%)	3 (50%)
**Civil status**		
Married	8 (100%)	5 (83.33%)
Widow	0 (0%)	1 (16.67%)
**Caregiver**		
None	4 (50%)	6 (100%)
Same age relative	3 (37%)	**–**
Younger relative	1 (13%)	**–**
**Living situation**		
With same age relative	8 (100%)	5 (83%)
Alone	**–**	1 (17%)
**Technology use**		
No use	4 (50%)	3 (50%)
Occasional	3 (38%)	3 (50%)
Daily	1 (12%)	**–**

*Age is stated in years. Other variables are stated in absolute number of individuals and percentage in brackets*.

### Effectiveness

Table 2 shows the effectiveness results, measured by the rate of users who were able to complete each task, and the average number of errors made by the participants in each task. At first, two users required help, reflecting that some usability problems still existed in the first high-fidelity prototype. Concretely, they did not understand the instruction given by the app. They were able to operate the devices once we helped them with the app.

**Table 2:** Effectiveness measurement results: task completion rate and number of errors.

**Variable**	**Task**	**First usability test**	**Second usability test**
**Task completion**	**Chair stand test**		
	Without help	5 (62.5%)	5 (83.33%)
	With help	2 (25%)	0 (0%)
	Unable to complete the task	1 (12.5%)	1 (16.67%)
	**Adapted Linda Fried's questionnaire**		
	Without help	3 (37.5%)	6 (100%)
	With help	5 (62.5%)	0 (0%)
	Unable to complete the task	0 (0%)	0 (0%)
	**Gait speed test**		
	Without help	5 (62.5%)	5 (83.33%)
	With help	3 (37.5%)	1 (16.67%)
	Unable to complete the task	0 (0%)	0 (0%)
	**Weight measurement**		
	Without help	4 (50%)	6 (100%)
	With help	4 (50%)	0 (0%)
	Unable to complete the task	0 (0%)	0 (0%)
Number of errors (mean)	Chair stand test	0.875	0.167
	Adapted Linda Fried's questionnaire	1	0.167
	Gait speed test	1	0.167
	Weight	0.625	0

*Task completion is stated in absolute number of individuals and percentage in brackets*.

Almost all of them were able to complete the tasks independently with the second prototype. It should be noted that, in both iterations, there was one user who was not able to complete the chair stand test. In the first case, this was due to a technical problem with the device not collecting data correctly. In the second case, the participant was distracted and did not hear the instructions indicating the start of the test, and then she did not know what she had to do and freely explored the application. Regarding the operation and handling of the hardware devices, we explained before starting the tests how to place them, switching them, and picking them up; later they were able to manage them adequately and performing the measurements.

With respect to the errors, in the test with the first high-fidelity prototype, test participants made one or fewer errors per task on average. With the second prototype test, participants made almost no errors.

### Efficiency

Table 3 displays the difference, in percentage, between the average number of taps made by the participants to complete the task and the optimal number of taps. These values have allowed us to assess the efficiency of the HMS. In the first prototype, users required 10 to 12.5% more taps than the optimal, except in the chair stand test, where participants got closer to the optimal (6.7%). In the second prototype, users only differed between 2.1 and 3.3%, except in the weight measure where test participants reached the optimal number of taps.

**Table 3:** Efficiency measurement results: Average deviation with respect to the optimal number of taps per task.

**Task**	**First usability test**	**Second usability test**
Chair Stand test	6.7%	2.8%
Adapted Linda Fried's questionnaire	11.3%	2.1%
Gait Speed test	10%	3.3%
Weight measurement	12.5%	0%

### Satisfaction

Regarding satisfaction, participants were asked to answer the SUS questionnaire. The obtained score barely varied in both evaluations: 84.06 (standard deviation 9.44) in the first high-fidelity prototype, and 83.75 (standard deviation 14.38) in the second one. Based on analysis of the answers to each of the questions, it can be deduced that participants considered the HMS easy to learn and use, and none of them found it unnecessarily complex or cumbersome. All the participants, except one, indicated that they would like to use the system frequently. Both SUS scores show a good usability level, above average for projects considered by SUS creators.

### Acceptability

Table 4 shows acceptability rates of the measuring devices. The gait speed and chair stand devices obtained over 85% in both tests. The weight scale, however, improved its acceptability rate in the second usability test, from 74.22 to 84.38%.

**Table 4:** Summary of the acceptability rates of the HMS obtained in the two usability evaluations involving older users.

**Task**	**First usability test**	**Second usability test**
Chair Stand test	88.28%	88.54%
Gait Speed test	85.94%	86.46%
Weight	74.22%	84.38%

Independently analyzing the answers to each question of the acceptability questionnaire, participants of the first usability test considered that both the gait speed and chair stand devices would motivate them to lead a healthier lifestyle (question 1), would make them feel more cared for (question 2) and would allow them to better control their health (question 4). However, none of the devices were considered a burden by test participants (question 3). In the case of the weight scale, participants also considered the device useful and helpful, but to a lesser extent.

In the second round of evaluation, results were similar, except for the weight scale, which obtained better results in all aspects.

Table 5 lists some of the positive answers provided to each of the acceptability questions in both evaluations. Table 6, in turn, lists the most relevant negative answers given by the participants.

**Table 5:** Positive answers to the acceptability open questions.

**Question 1: What are the main problems you found when using the system?** *“None.”* *“I do not find any problems.”*
**Question 2: How did you like the system in general?** *“It is very good.”* *“It is very simple to use after the first try.”* *“Each task is easier.”* *“I like it a lot because it helps me always be in touch with my doctor.”* *“It is very useful, as I will no longer have to remember everything when visiting the doctor, since the system will store it.”* *“I am entertained for a while.”* *“It is not hard work.”*
**Question 3: How did you feel when you used the system?** *“I felt comfortable, and it does not require too much effort.”* *“I have not felt bad at all.”* *“Comfortable.”* *“It is a help for me.”*

**Table 6:** Negative answers to the acceptability open questions.

**Question 1: What are the main problems you found when using the system?** *“I am used to going to my nurse to weigh me and I like it. I do not want to change this.”* *“The task of the questions. I have not understood them well.”* *“Too much reading. I would prefer single words.”* *“At the beginning, I doubted that I could use it.”*
**Question 2: How did you like the system in general?** *“I think I would prefer to do it with my doctor because she motivates me while I am doing the task.”* *“I think it is too much work to do all these tasks every morning.”* *“The doctor can know if I am not doing what she has asked me to do.”*
**Question 3: How did you feel when you used the system?** *“I have not needed it, but I think there should be something to ask for help.”* *“Good, except in the task of the questions.”* *“Nervous because of the reading, because I don't read very well.”*

### Usability Analysis

After the analysis of the data (both quantitative and qualitative) obtained in the first usability tests, six major usability problems were detected. Along with each problem we present the solution implemented in the second high-fidelity prototype, which proved effective in the second usability test.

#### Excessive Text in the Screens

Users were mostly able to read and understand instructions in the interaction flow. However, when facing long blocks of text, they showed tiredness and tended to skip lines. Therefore, they might be missing some relevant information for the correct performance of the self-assessment.

**Figure 3:**
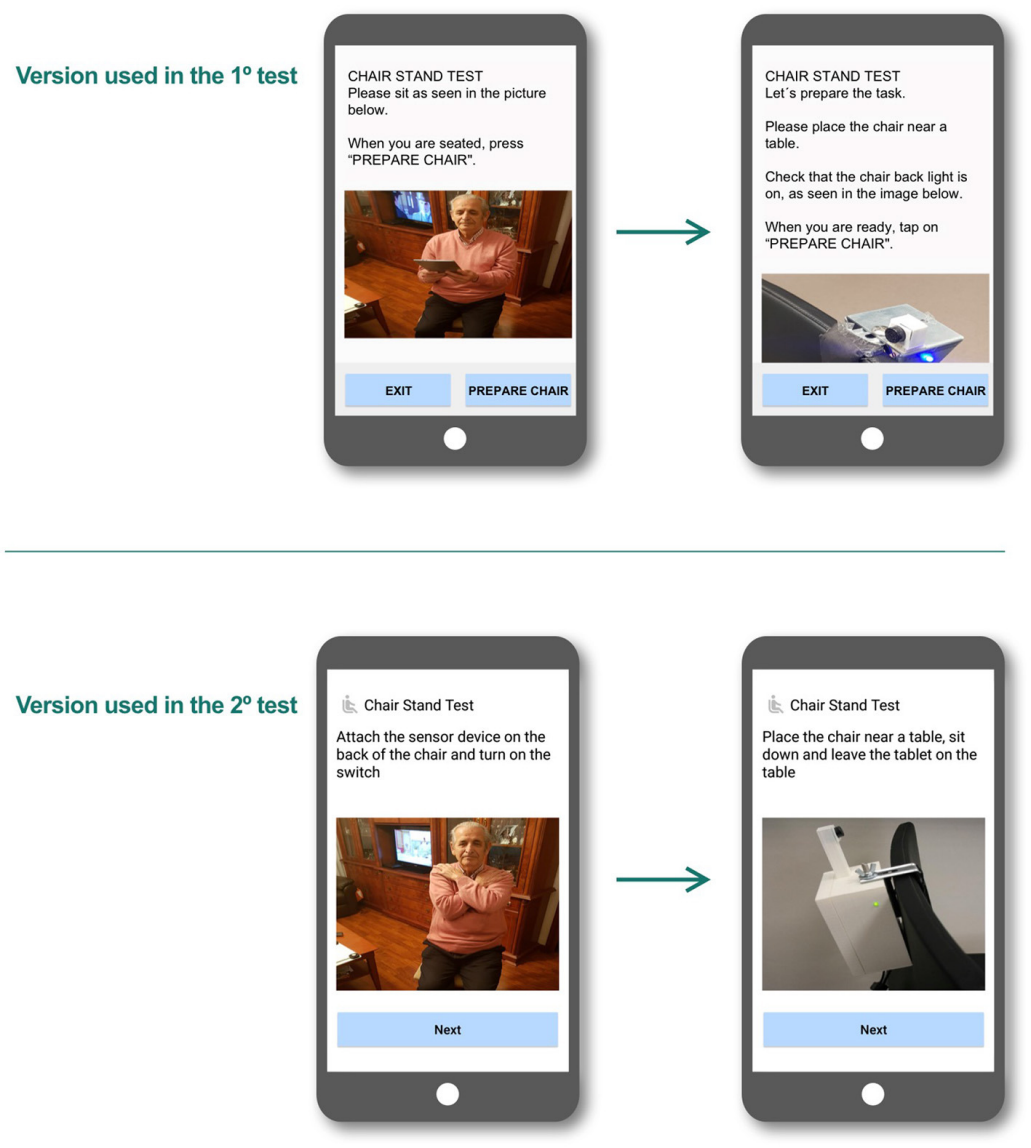
Changes in the mobile app to reduce text.

To overcome this problem, textual instructions were shortened, removing all dispensable content, and was complemented with audio instructions (see Figure 3).

#### Response to Incoming Notifications

Users had problems reacting to incoming notifications. The prototypes in the study used notification channels provided by the operating system. This does not allow designers to choose a type of notification which older persons could easily understand and react to.

Despite acting in response to the incoming acoustic signal, most test participants required assistance to identify the notification message, which appeared as a button at the center of the screen. They specifically commented on the small size of the font. Furthermore, very few subjects were able to tap twice quickly enough, which is the operating system gesture to open the notification. In general, single tapping should be used throughout the interaction design, for the sake of consistency.

**Figure 4:**
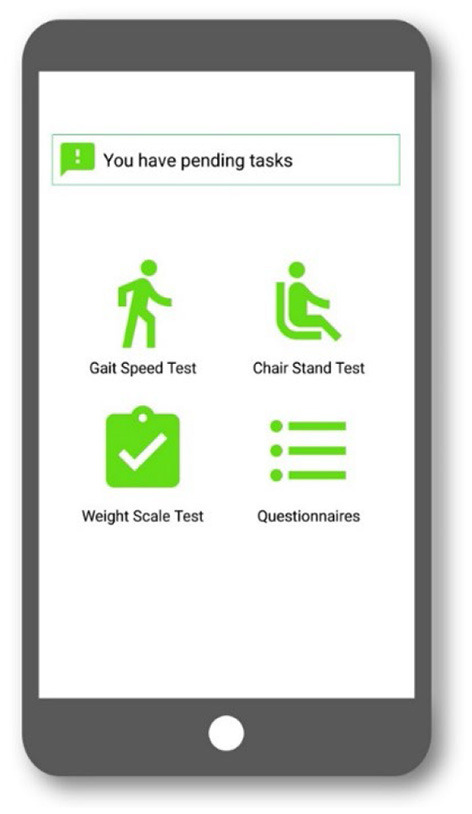
Data capture app dashboard in the second test.

This usability problem was overcome by the removal of the incoming notifications. Instead, users will access the different tasks by opening the application and accessing the prescribed task from a dashboard which acts as the home screen, where pending tasks are represented with green icons, and completed tasks with gray icons. The resulting dashboard is shown in Figure 4. This new method requires an additional step from users but reduces the confusion they experienced.

#### Text in the Bottom Part of the Screen

**Figure 5:**
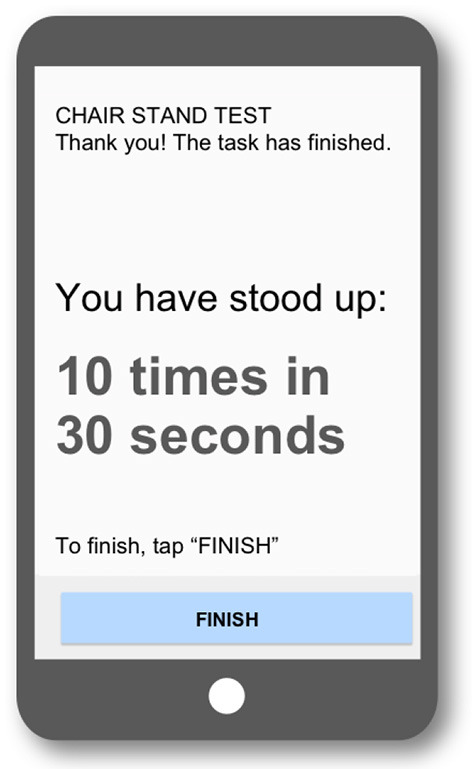
Example of chair stand test result where the users did not react to the instructions at the bottom.

Users had problems reading text at the bottom of the screen. They started reading the text block at the top and often missed the instructions above the navigation buttons (see Figure 5).

This usability problem was solved by removing any text giving instructions in the bottom part of the screen.

#### Response to Pop-up Messages

In the interaction design in the first mobile app version, some screens included a confirmation step, which was implemented via a pop-up message. Most users did not notice the emergence of that message and expected to navigate to a new screen. This was the case on the questionnaire screens. Users expected the app to move forward to the next question, but this transition required the user to tap the “Next screen” button. Users did not react to the pop-up message and believed the transition had already taken place, reading the same question, and even answering it again.

**Figure 6:**
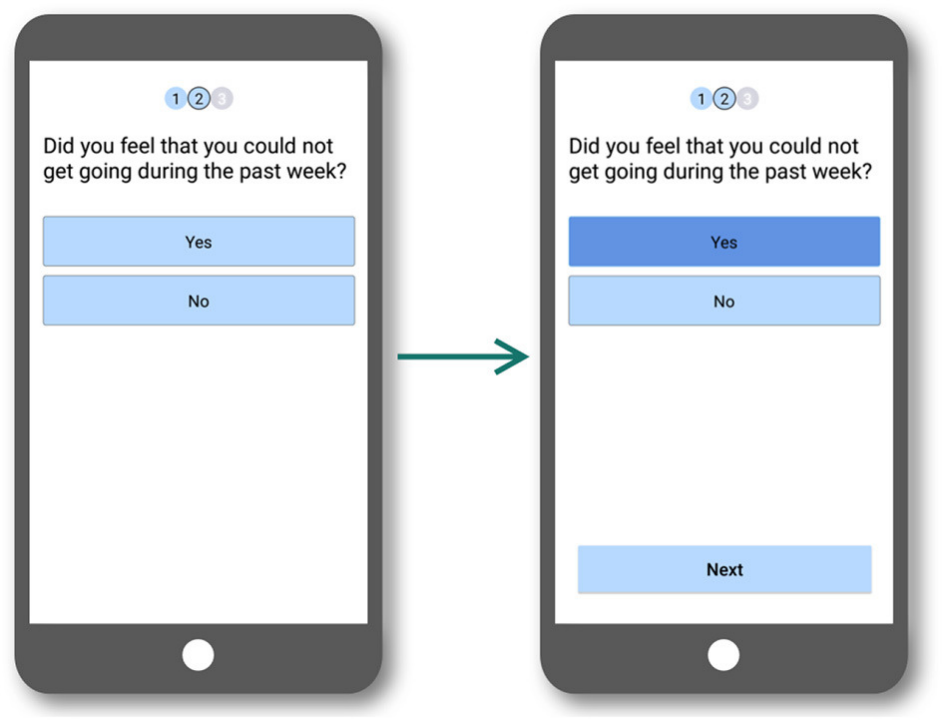
Interaction flow for the questions used in the second test where pop-up messages were removed.

This problem was solved by removing confirmation messages via pop-up. Screens in the new design do not have any modal messages to avoid users thinking the context has changed. Therefore, when they answered a question, the response was marked, and the user was then allowed to continue to the next screen (see Figure 6).

#### Complex and Inconsistent Navigation

In the interaction design of the first version of the app prototype, users were able to exit the current task with three different buttons, located in different parts of the interface, depending on the step of the task.

**Figure 7:**
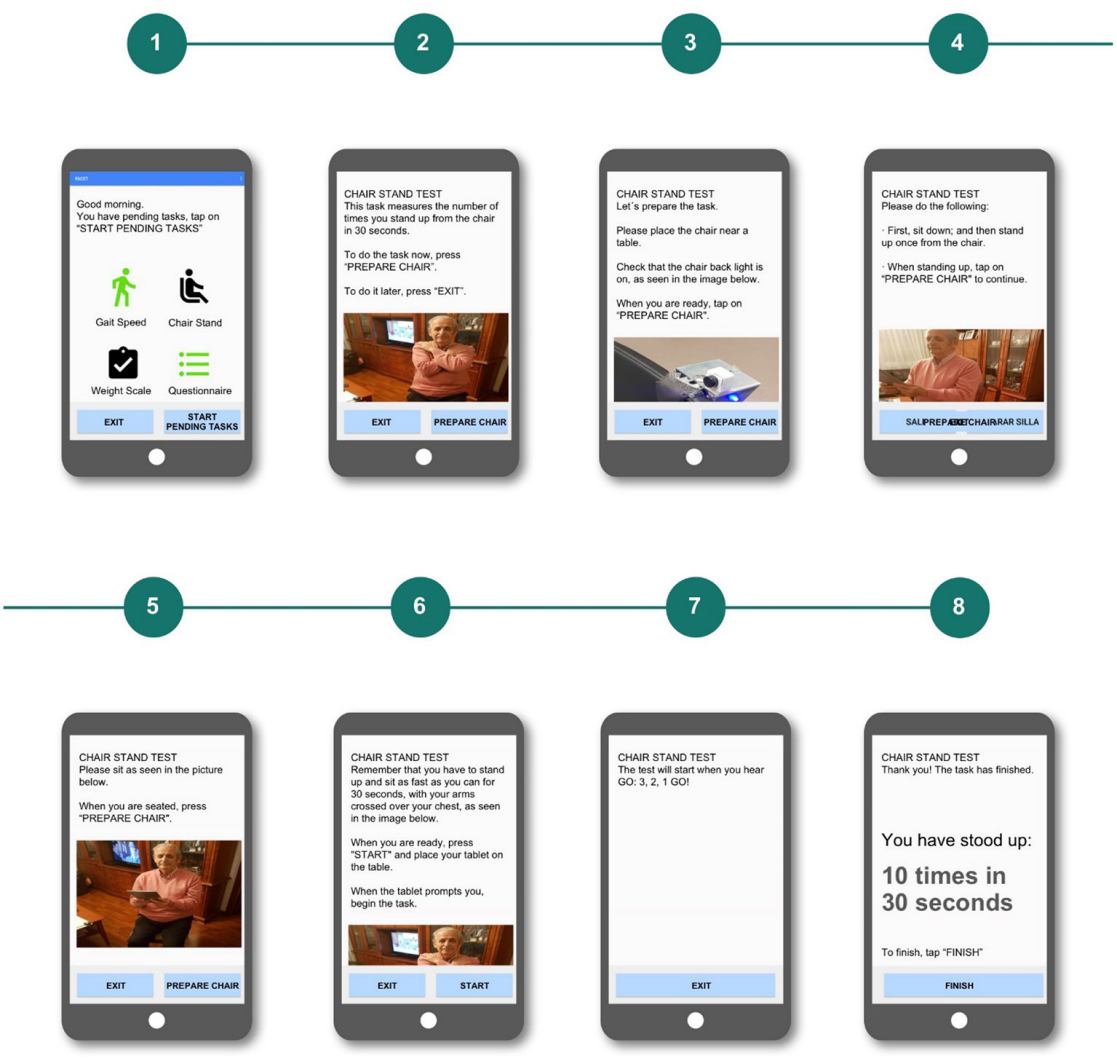
Chair stand test used in the first test.

At the main screen of every task, an “Exit” button appeared in the lower-right corner of the screen. Having different ways to navigate to the main screen was confusing for test participants (see Figure 7).

In addition, some other test participants were confused in the questionnaire task because of the existence of two navigation buttons that allowed them to go to the previous (“Previous question”) or the next (“Next Question”) screen. This degree of freedom was confusing, as it did not match their mental model. Older participants expected linear and unidirectional processes, so the possibility of moving back and forth was overwhelming for them.

**Figure 8:**
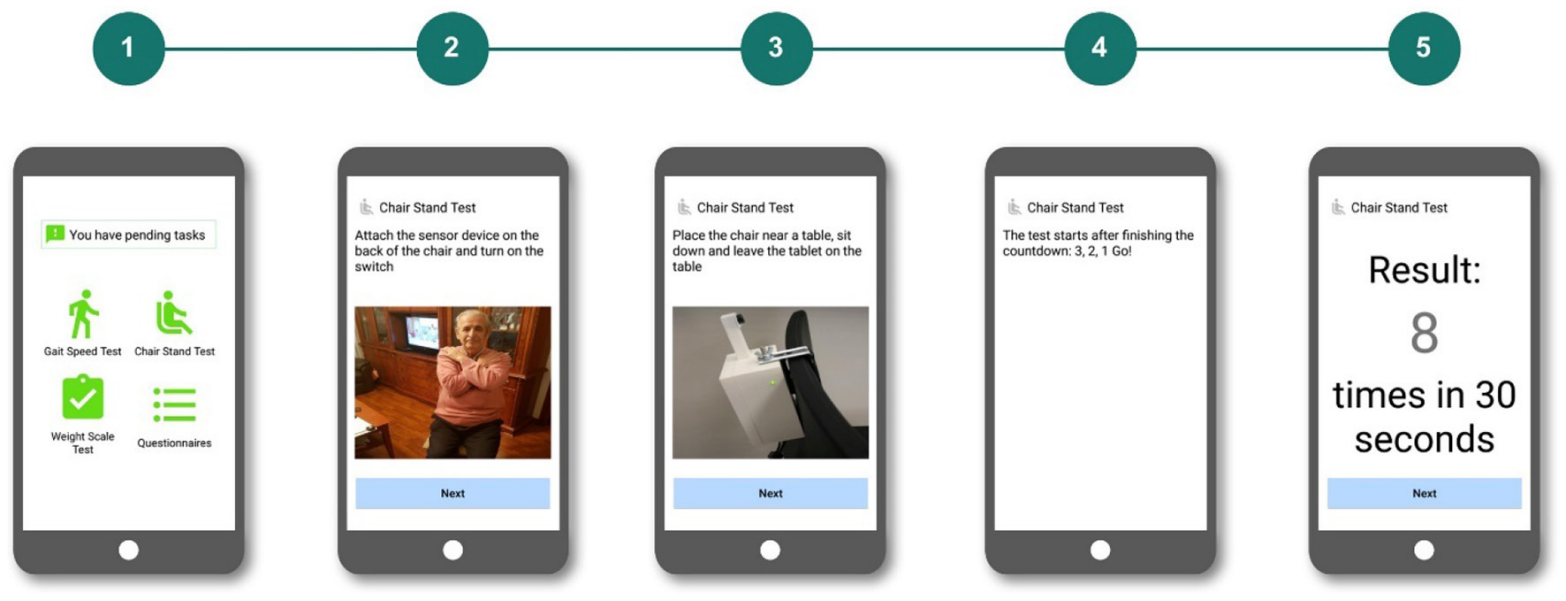
Chair stand test interaction flow used in the second test, where the user only had one navigation button.

The solution to this problem was to provide a simpler and more consistent navigation. Accordingly, the new design had only one navigation button in each screen, only allowing users to move forward to the next screen and always with the same label. Users then have to either complete the whole task or close the application and start over (see Figure 8).

#### Involuntary Multiple Tapping

Some users moved several screens ahead with a single tap gesture. Older persons often lack soft-movement skills. In some cases, when they tried to tap once a button, they were tapping twice or even three times on the same area of the screen. We tried to build a consistent interaction model in which the “next” button in consecutive screens is typically positioned in the right-bottom area. Therefore, if several consecutive taps were applied in the same area, users would move forward unintentionally due to their inability to perform precise movements.

This usability problem was overcome by incorporating a lapse of time when a new screen is reached, during which the system ignores any action from the user. The purpose of this tactic is the prevention of unintended multi-tapping, so users have time to recognize the transitions between screens and do not miss their content.

All these problems were addressed and solved as part of the new interaction design for the mobile app that was evaluated in the second usability test.

No major usability problems were identified in the second usability test. Therefore, we considered the system ready for clinical evaluation.

## Discussion

Usability and acceptability are very relevant aspects in any software system, but they become paramount in the case of older users, as stated by Fisher et al. (37), and in the reviews presented by Yusif et al. (38), and Holthe et al. (39). Thanks to user-centered design, our solution meets the needs of older users by means of a good UX, including high usability and acceptability results. Mobile health often fails due to lack of adoption, which is highly correlated to low levels of usability and acceptability (40). Recently, other related works are also including a user-centered approach to enhance usability (41), showing a changing tendency in the implementation of such systems.

The main recommendations for the design of mobile applications for our target group, stemming from our usability study, are as follows:

- **Textual instructions should be as concise as possible**. We included lengthy texts in our user interface because we tried to faithfully reproduce what physicians told their patients when administering the CGA. Physicians tend to provide more information than needed, to make sure that patients understand properly. This strategy does not work for the human-computer interaction with older users, since they may easily tire of reading. Even if the intention was to make the instructions clearer by being more detailed, the effect was just the opposite. Androutsou et al. identified the difficulty older users have reading text in notifications (42).
- **Use audio whenever possible to supplement text notifications**. Due to the common occurrence of eyesight problems in older users, audio helps with problems reading text.
- **Simple and consistent navigation**. We finally opted for just one navigation button in each screen. Even if the user has less freedom to navigate, user errors diminish. Reducing complexity in user interfaces is a successful strategy with this kind of users, recommended by Fisher et al. (37).
- **Pop-up messages and modal notifications should be avoided**. They may be interpreted as transitions to a different screen by the older user. Alternatively, more screens can be added, even at the risk of adding steps to the task. This recommendation goes in line with the advice from Fisher et al. about removing layered windows that challenge memory/motor function (37).
- **Provide guided navigation with less freedom**. Older users have more difficulties coping with complex navigation schemes, especially for users unaccustomed to technology. User errors diminish with guided navigation, even if efficiency is lowered. We are violating Nielsen's usability heuristic of “User control and freedom” (43), but freedom may overload the cognitive processes of older users when using a mobile application, since they may have more difficulty developing an appropriate mental model for the app navigation. This recommendation contributes to the overall aim of reducing complexity, as mentioned above.
- **Include timeouts to avoid multiple tapping**. Accidental tapping twice in the position of a control can be avoided if a timeout is set before a new interaction in the same place is allowed.

Users provided positive feedback about the HMS, remarking that it was easy to use and expressing that it would be useful for better management of their health. They also highlighted the negative effect the HMS may have on the relationship with their physicians, in the sense that they would be visited by their physicians less often. This fear has already been described in the literature (44).

Due to the heterogeneity in skills and education of the older population, an adaptation of the HMS to different user profiles might be desirable. Strategies such as the automatic logging and analysis of events and errors occurring during the use of the HMS (45), in combination with the categorization of users regarding skills, capabilities, and preferences may shed light on the appropriate interaction processes for each older user. This approach has already been used in multiple applications and automatic usability evaluation methods that have been empirically validated (46).

Regarding limitations of the study, we can mention two: First, 14 participants may not include all relevant diversity among the older adults. Second, patients were recruited from a database of volunteers, who might be more enthusiastic than the general population. In our sample, although most of the participants reported that they were not regular mobile users, we need more diverse users to confirm our findings.

We carried out these usability tests as part of our participatory design effort, aiming to get insights about the problems our potential users may face. In the Human-Computer Interaction field, it is not necessary to achieve statistical significance in this kind of study, as it would be required for controlled experiments. Dumas and Redish state that a typical usability test includes 6 to 12 participants in two to three subgroups, since 90% of usability problems may be identified with 10 participants (47). Regarding scientific papers, a study presented at the CHI-2016 conference (the most important conference in the field) acknowledged that the most common sample size was 12 subjects in papers accepted for an earlier edition of the conference (48). Even if there is not one sample size that fits all research projects, a sample size of 14 subjects is common in studies not looking for statistical significance, like ours.

## Conclusions and Future Work

The design of any mHealth system for an older population is challenging. A UCD process is the best way to adequately cater to the needs of this segment of users. Our iterative approach allowed us to identify the main usability problems in the first high-fidelity prototype of our HMS system.

Results show that the older adults who participated in the usability tests considered the final version of our system easy to use and effective, without affecting their satisfaction. Acceptability results are also very promising.

Older adults can be empowered and contribute to a proactive and predictive frailty care model autonomously. We have demonstrated the feasibility of an adapted CGA in the community dwelling through mHealth.

The outcomes of this research open new perspectives in the management of frailty. However, further research must be carried out to investigate the feasibility, clinical impact, and cost-effectiveness of an intervention based in our HMS.

The implementation of motivation strategies and the development of more human user interfaces might facilitate the adoption of technology by the elderly. Education and dissemination efforts should be also carried out to raise awareness among elderly populations about the potential benefits of this kind of intervention, thus reducing their reluctance.

Finally, further research also includes a randomized clinical validation of the HMS in the frame of the FACET project. Patients will use the system at home for 3 months. After that, we will take the system further to product design and needed marking.

## Data Availability Statement

The raw data supporting the conclusions of this article will be made available by the authors, without undue reservation.

## Ethics Statement

Before starting the study, the ethical approval was requested and obtained from the Clinical Research Ethics committee of HUG on 29th October 2017, with number 17/72. The patients/participants provided their written informed consent to participate in this study.

## Author Contributions

EV-M and XF have designed the usability study and interpreted the results. CM has performed the quantitative analysis. EV-M, RP-R, MV-A, and AS-S have performed the usability tests, acting as: facilitator EV-M, MV-A note taker and dataset creator, RP-R and AS-S as technical support and observers. LR-M is the principal investigator and performs the quality assurance. They all have contributed to the writing and editing of the paper. All authors contributed to the article and approved the submitted version.

## Conflict of Interest

The authors declare that the research was conducted in the absence of any commercial or financial relationships that could be construed as a potential conflict of interest. The reviewer, RIG-B, declared a shared affiliation, though no collaboration, with several of the authors, EV-M, XF, CM, and AS-S, to the handling editor.
