# Supplementary material for: Home Monitoring System for Comprehensive Geriatric Assessment in Patient's Dwelling: System Design and UX Evaluation
Source: Front Digit Health. 2021 May 7;3:659940. doi: 10.3389/fdgth.2021.659940 (PMC8521833; doi:10.3389/fdgth.2021.659940)
Supplement: Supplementary file 1 [file Data_Sheet_1.docx]

# Appendices

## Acceptability questionnaire

Questions for each of the three external devices:

1. The information it provides would help me having a healthier lifestyle
2. It would make me feel more cared for
3. It would be a burden for me
4. It would allow me to control my own health

Answers: 5 Likert scale from fully disagree (1) to fully agree (5).

Acceptance formula (%):

$$accep= 100\cdot\frac{\mu\left(\left(p1-1\right)+\left(p2-1\right)+\left(p4-1\right)+\left(5-p3\right)\right)}{4}$$

Three ad-hoc open questions for overall system acceptance:

1. What are the main problems you found while using the system?
2. How did you like the system in general?
3. How did you feel when you used the system?

## Adapted Linda Fried’s Frailty Criteria

| **Frailty Criteria** | **Classical Assessment (extracted from Fried et al.** [5]**)** | **Adaptation in Home Monitoring System** |
| --- | --- | --- |
| Weight Loss | Self-reported. Answer to one Yes/No question:  *“In the last year, have you lost more than 10 pounds unintentionally (i.e., not due to dieting or exercise)?”* | Automatically measured using the weight scale of the HMS. |
| Exhaustion | Self-reported. Answers to two Likert-based questions:   1. *How often in the last week did you feel that everything you did was an effort?* 2. *rarely or none of the time (<1 day)* 3. *some or a little of the time (1–2 days)* 4. *a moderate amount of the time (3–4 days)* 5. *most of the time* 6. *How often in the last week did you feel that you could not get going?* 7. *rarely or none of the time (<1 day)* 8. *some or a little of the time (1–2 days)* 9. *a moderate amount of the time (3–4 days)* 10. *most of the time* | Self-reported. Answers to two Yes/No questions through the mobile application of the HMS.   1. *Have you felt everything you did was an effort during last week?* 2. *Have you felt during last week that you could not get going?* |
| Walk Time | Time required to walk 15 feet. Measured with a stopwatch by a healthcare professional and stratified by gender and height. | Automatically measured using gait speed device of the HMS. |
| Physical Activity | Energy consumption (in Kcals/week) stratified by gender. | Self-reported. Answer to one Yes/No question through the mobile application of the HMS.  *Have you done more than 2 hours of physical activity in the last week*? |
| Grip strength | Grip strength (in Kg) stratified by gender and body mass index. | Automatically measured using chair stand device of the HMS |

## Final mobile application interaction flows

This appendix includes the interaction flows for the final version of the mobile application, the one used in the second usability test.

1. Chair stand test is included in Figure 8
2. Adapted Linda Fried’s questionnaire
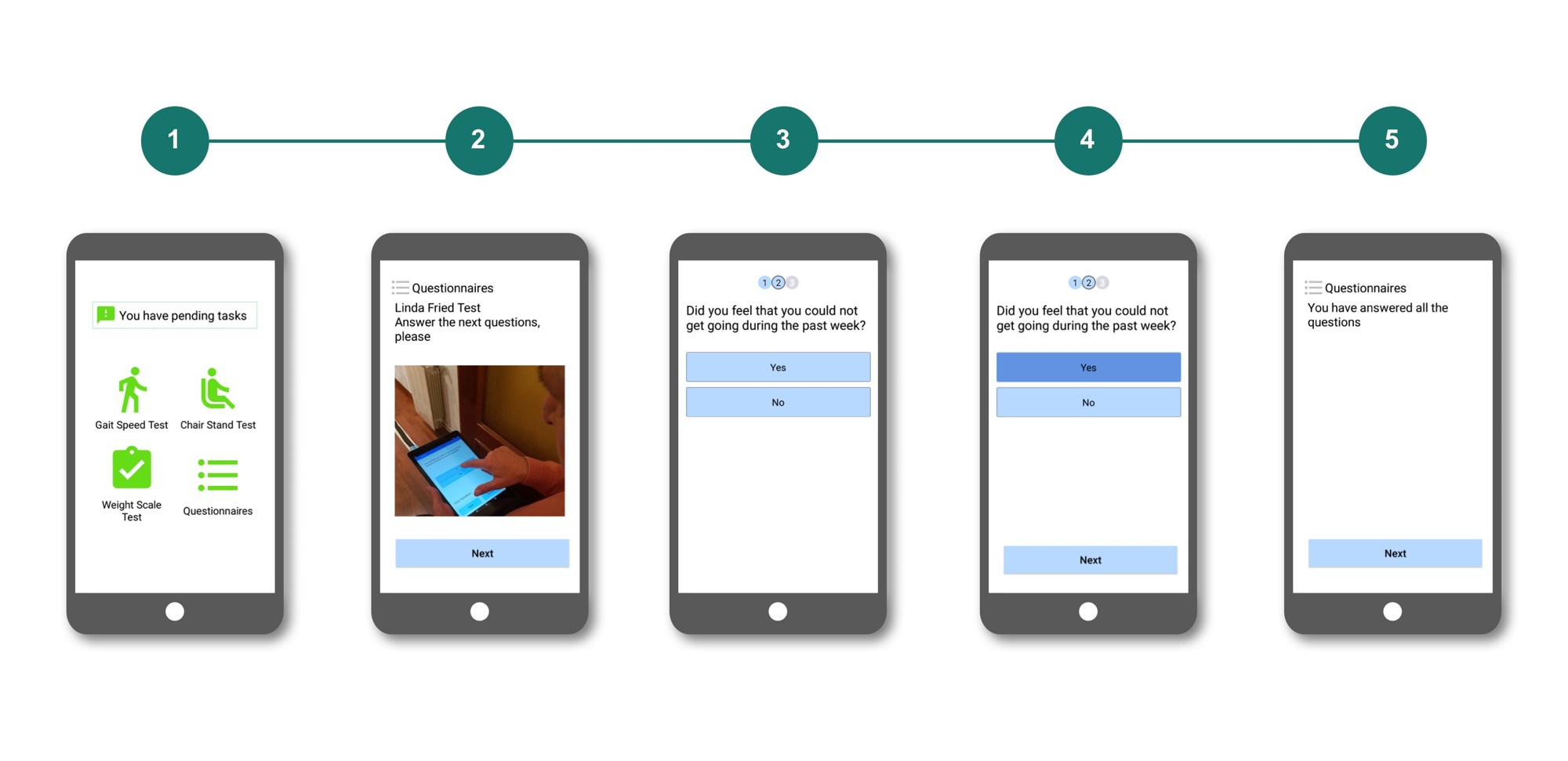


Figure 9 Adapted Linda Fried's questionnaire

1. Gait speed test


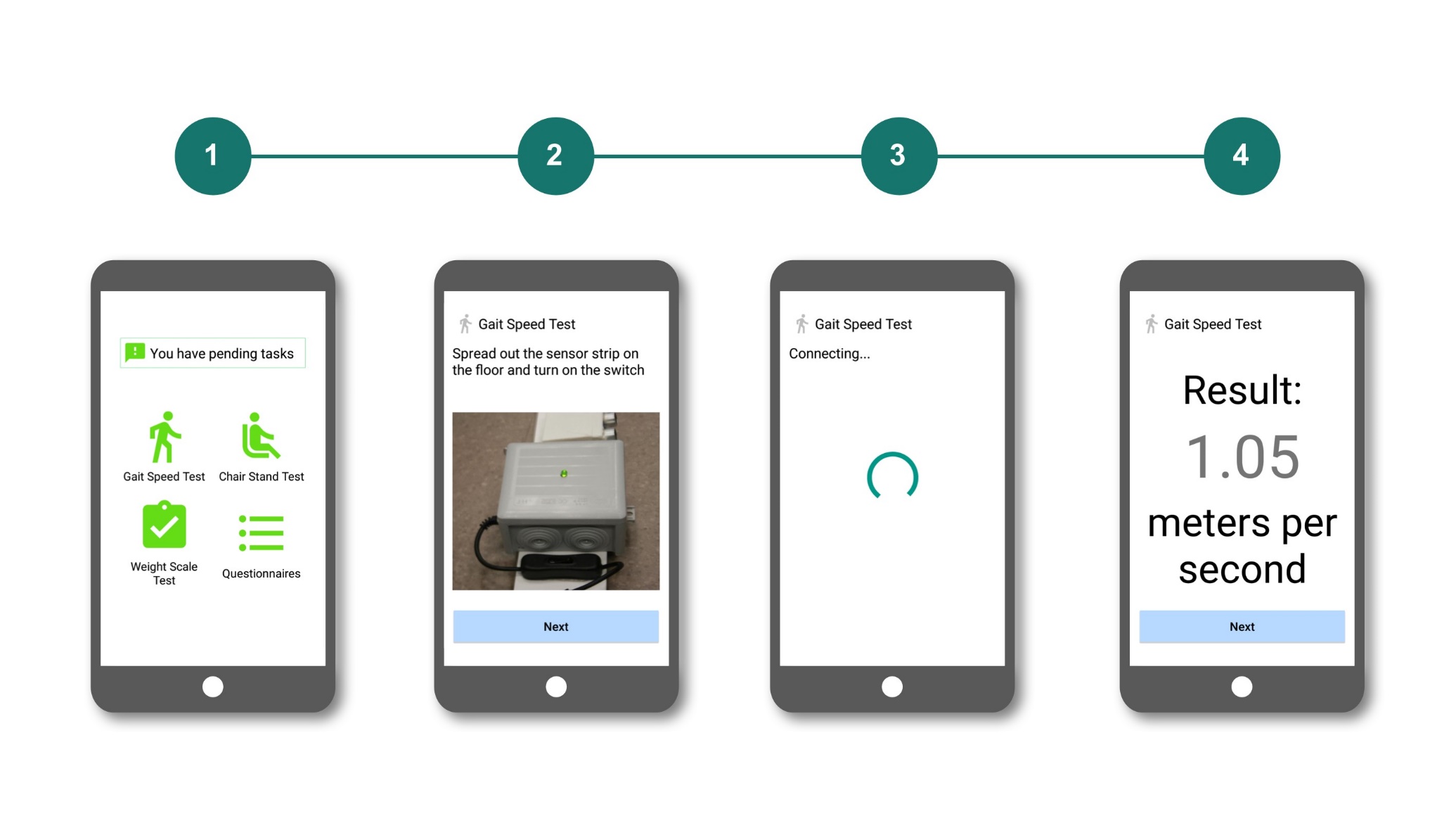


Figure 10 Gait speed test

1. Weight measurement


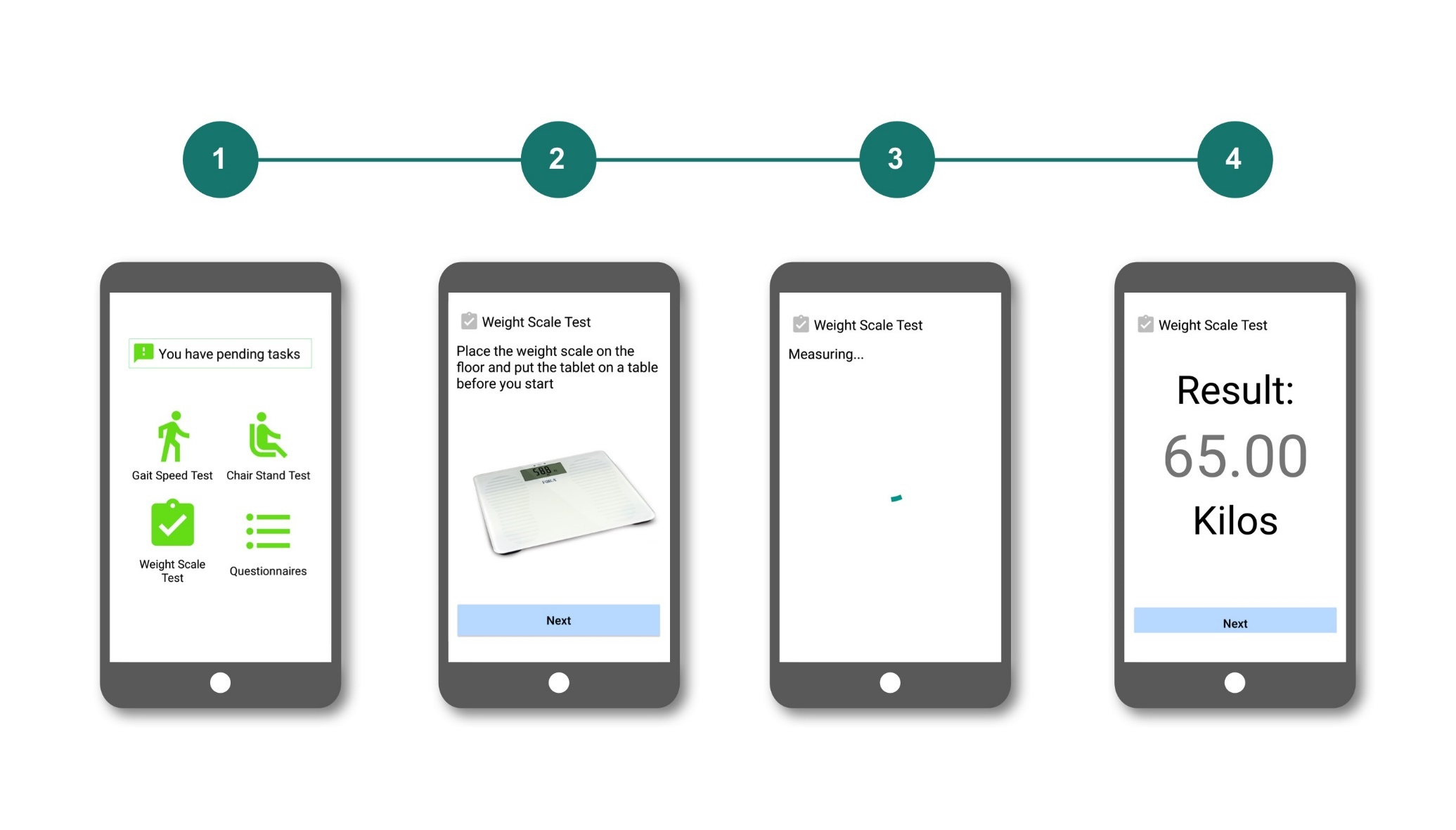


Figure 11Weight measurement
